# Training obstetrics and gynecology residents to be effective communicators in the era of the 80-hour workweek: a pilot study

**DOI:** 10.1186/1756-0500-7-455

**Published:** 2014-07-17

**Authors:** Omar Maurice Young, Kristiina Parviainen

**Affiliations:** 1 Division of Maternal-Fetal Medicine, Department of Obstetrics, Gynecology and Reproductive Sciences, Magee-Women’s Hospital/ University of Pittsburgh Medical Center, 300 Halket Street, Pittsburgh, Pennsylvania 15213, USA

**Keywords:** Role-playing, Professionalism, Physician-patient relationship, Simulated patient encounters, Obstetrics and gynecology, ACGME milestones

## Abstract

**Background:**

To ensure optimal patient care, physicians must establish effective patient-physician relationships and thoughtfully incorporate their patients’ perspectives into their counseling. Historically, these skills are acquired with increasing clinical experience. However, given increasing work-hour restrictions, OB/GYN residents have fewer opportunities to develop these skills. Therefore, the objective of this study was to determine if an interactive learning method is an effective tool by which to teach OB/GYN residents how to communicate with complicated patients.

**Methods:**

An experiential simulation model was developed to teach OB/GYN residents effective communication skills for dealing with patients experiencing a pregnancy-related complication. A simulated patient interaction was designed for first-year residents. Specific scenarios were constructed based on challenging clinical scenarios identified by second-year residents. Non-judgmental communication, culture competency awareness and reflective listening were key skills that were taught as part of the clinical scenarios. Both acceptability and utility of the exercise with the first-years was assessed by a follow-up survey.

**Results:**

Seven first-year residents participated in the education session consisting of four physician-patient interactions with specific learning objectives for each. These first-year residents all indicated that they would employ the skills practiced during the intervention into their future practice of medicine, and that their comfort level in caring for complex obstetric patients had increased. Moreover, all first-year residents endorsed that this educational strategy was potentially applicable to other aspects of their training.

**Conclusions:**

Simulated patient exercises can be utilized in multiple arenas to teach OB/GYN residents communication skills, while simultaneously addressing their clinical knowledge deficits. Early implementation of such a curriculum in an OB/GYN residency will lay the foundation for the development of empathetic and culturally competent physicians.

## Background

With the introduction of resident work hour restrictions by the Accreditation Council of Graduate Medical Education (ACGME) in 2003 and subsequent modifications in 2011, there is a growing debate among the medical and surgical specialties regarding how to most effectively train residents to become fully independent practitioners with less time [1]. The apprenticeship model of medical education in the setting of current duty hour restrictions is increasingly challenging. In this current environment of restricted resident work hours, physician-educators must strive to balance formal didactic and hands-on training. A recent survey of physician-educators in obstetrics and gynecology (OB/GYN) by Espey et al. reported concerns regarding the negative impact of resident work hours on resident education [2]. Nearly 63% of the respondents felt that the global education of an obstetrics and gynecology resident was worse, as compared to what it had been prior to the implementation of work hour requirements by the ACGME [2]. With the implementation of the 16-hour workday restriction for first year residents, senior level general surgery residents (94% of second- through fifth-year residents) expressed that such restrictions would adversely impact the education of their more junior counterparts [3].

The aforementioned problem is exacerbated in OB/GYN, as residents have a vastly broad body of knowledge and technical expertise to master in a 4-year span under the umbrella of restricted work hours. At the conclusion of an OB/GYN residency, the resident is expected have a broad knowledge of generalist topics; however, this may come at the expense of having only cursory knowledge of the sub-specialties of family planning, reproductive endocrinology and infertility, gynecologic oncology, uro-gynecology and maternal-fetal medicine (MFM). In the management of an obstetric patient, however, a seemingly routine case within the scope of practice of a generalist OB/GYN can quickly evolve into a more complex maternal-fetal issue, with the patient expecting her doctor to manage the situation with expertise. Given the unpredictable frequency of these clinical situations for trainee participation, simulations for such scenarios as shoulder dystocia and operative vaginal delivery continue to evolve not only for clinical skills development but also for competency assessment.

“Role-playing”, or simulated patient interactions, have been applied in various aspects of medical education, particularly in the education of medical students [4]. In a British study looking at the benefits of role playing on medical student education, of the 274 students responding to a post-role playing assessment, 96.5% found the session to be helpful to allowing them to develop their skills, receive feedback and obtain perspective from other participants [5]. Numerous suggestions were offered by those that participated regarding how the role-play could have been even more effective. These included increased time to role-play, increased personal feedback, and making scenarios as close to real-life situations as possible [5]. Nevertheless, it is unclear as to whether or not such an educational tool can be successfully applied to the education of OB/GYN residents. Thus, we propose an innovative and experiential approach at our institution to teach OB/GYN residents effective communication techniques utilizing complex obstetric scenarios relevant to their daily clinical responsibilities. We hypothesize that such an approach will enable OB/GYN residents to become more facile in communicating with even the most difficult patients under the most complex of circumstances and that a simulated patient exercise would be a means by which to accomplish this. Because junior residents are often the initial faces of the care team, it is important for residents to commence the acquisition of these skills early on in their training.

## Methods

We developed a simulated patient exercise at Magee-Women’s Hospital of University of Pittsburgh Medical Center, which is a tertiary care center with greater than 10,000 deliveries annually and a busy perinatal referral service for maternal-fetal medicine. As part of formal resident didactic education at our institution, first-year residents participate in “Intern Ed”, a separate five-hour session once per month dedicated to level-specific skill building. These skills include emergency/crisis management, placement of long-acting forms of reversible contraception and the navigation of issues of systems-based practice. We utilized the designated two-hour session for patient counseling for our simulation exercise.

In preparation, we e-mailed the current second-year residents with a link to an on-line survey with questions querying their readiness to handle various obstetrical situations commonly encountered on a labor and delivery unit, based on their experiences as interns. The content of the survey may be found in Additional file 1. As part of the survey, we asked second-year residents (PGY2) to review a list of common complicated obstetrical scenarios as noted in Table 1, including: peri-viable preterm premature rupture of membranes, counseling for a trial of labor after a cesarean delivery, and refusal of blood products by a Jehovah’s witness. Using a 7-level Likert scale, we asked second-year residents to rank these scenarios from “most comfortable” to “least comfortable” in terms of how confident they felt in their personal ability to provide the requisite counseling. These choices were pre-selected by the investigators as clinical issues commonly encountered by junior residents at our institution. Based on these results, we developed four scenarios for simulation, from among the ones identified by the PGY2s as the “least comfortable”. These scenarios can be seen in Additional file 2. Second-year residents did not undergo the simulated patient exercise.

**Table 1:** Second-year resident comfort level with counseling patients with complicated obstetrical issues

**Obstetrical scenario**	**1 “Most comfortable”**	**2**	**3**	**4 “Neutral”**	**5**	**6**	**7 “Least comfortable”**
A	2 (22%)	1 (11%)	0 (0%)	2 (22%)	2 (22%)	1 (11%)	1 (11%)
B	1 (11%)	3 (33%)	3 (33%)	1 (11%)	1 (11%)	0 (0%)	0 (0%)
C	2 (22%)	0 (0%)	1 (11%)	4 (44%)	2 (22%)	0 (0%)	0 (0%)
D	3 (33%)	1 (11%)	4 (44%)	0 (0%)	0 (0%)	1 (11%)	0 (0%)
E	0 (0%)	0 (0%)	1 (11%)	2 (22%)	2 (22%)	0 (0%)	4 (44%)
F	0 (0%)	5 (55%)	0 (0%)	0 (0%)	1 (11%)	3 (33%)	0 (0%)
G	0 (0%)	0 (0%)	0 (0%)	0 (0%)	1 (11%)	4 (44%)	4 (44%)

Table Legend: This table demonstrates the frequency of second-year residents who described if there were comfortable with counseling patients with complicated obstetrical issues according to specified scenarios. Nine residents ranked the scenarios on a Likert scale from “1” for “most comfortable” to “7” for “least comfortable”. Frequencies of responses are expressed as the raw number and the percentage in parenthesis. The scenarios were as follows:

A. Counseling a woman at 18 weeks on all her management options who has experienced preterm premature rupture of membranes (PPROM)

B. Management options for a woman at 28 weeks who has experienced preterm premature rupture of membranes (PPROM)

C. Counseling a G1P0 at 20 weeks with a cervical length of 17 millimeters

D. Discussing the risks and benefits of a repeat cesarean delivery versus trial of labor after cesarean delivery

E. Counseling a woman at 26 weeks with a heavy bleeding episode on management options after you have diagnosed her with a complete placenta previa

F. A nulliparous diabetic patient presents to your clinic at 36 weeks for a prenatal visit. Her fetus is breech. She desires an ECV. You go through the chart and see that her ultrasound yesterday indicates an EFW of 4400 g.

G. A woman has an ultrasound highly suspicious for a placenta accreta. She desires to keep her uterus.

Prior to the Intern Ed session, we sent all current first-year residents a separate on-line survey to assess their past experiences with simulated patient interactions. We also shared with them details of our proposed educational module. This content may be found in Additional file 2.

In order to ensure sufficient clinical knowledge given the timing of the session during the academic year (early October 2013), we e-mailed all first-year residents one week prior to the simulation with formative articles related to the clinical scenarios to be addressed in the simulation [6-9]. Prior to starting the simulation exercise, we set the following expectations and ground rules: 1) Treat each scenario as an actual clinical encounter; 2) Option to pause the scenario at any time to ask questions; 3) Anything occurring or said in the room would remain confidential. We divided our cohort into groups of two to three residents. For each scenario, we provided each group with a sealed, opaque envelope with explicit instructions for the role of “patient”, “physician”, or “family member”. The groups were then provided five minutes to strategize how they would approach their scenario. While the scenarios provided an opportunity to apply knowledge of complicated obstetrical issues to simulated cases, they were targeted to promote practice of key skills in establishing an effective physician-patient relationship:

a. Non-judgmental communication: ensuring that personal bias does not enter into a conversation with a patient to influence clinical decision-making [10];

b. Culture competency awareness: appreciating how the nuances of a patient’s belief system influences their health care decisions and a physician’s counseling of that patient [11]; and

c. Reflective listening: listening to what the patient is saying and then repeating what he or she has said so as to confirm that one has understood [12].

Once each group had designated an actor for their respective roles, the actors simulated the obstetrical scenario in front of the entire room. Initially the residents played out each scenario until its completion, without interruption. Then, we asked the participants how they felt playing their individual roles and if they would have changed anything. In the discussions, we linked the events of the simulated patient exercise with the readings provided prior to the intervention to broaden participants’ clinical knowledge base. At the end of each scenario, we asked the collective group to answer the following questions:

1. What were the challenges of this scenario?

2. How else could you have approached this scenario?

3. If you have been confronted with this scenario, how did you manage this?

4. What are the take-home points from this scenario?

The actual scenarios used can be found in Additional file 3. After the conclusion of the simulation, all participants were asked to complete a follow-up survey to evaluate the acceptability and the utility of the exercise for their overall education. We tabulated descriptive statistics for our survey questions via STATA 12.0 (StataCorp, College Station, TX). This education research study qualified for exempt status as determined by the University of Pittsburgh Institutional Review Board, #PRO13070456, on August 16, 2013.

## Results

### Second-year residents

Our second-year class is comprised of ten residents, including nine women and one man, whose ages range from 27–36 years. Nine out of ten (90%) second-year residents responded to the second year survey. Six (67%) of 9 second-year residents responded that their experiences as a first-year resident only *somewhat prepared* them to effectively communicate with high-risk obstetrical patients. Five (55%) of the respondents stated that they *felt intimidated* when asked to evaluate a MFM patient in OB Triage or on the antepartum floor. Eight of the nine (89%) second-year respondents reported that either journal clubs or lectures were the least effective methods by which to learn new material. The majority of second-year residents (77%) reported that case-based learning was the most effective way to learn new material. Only one of the respondents indicated that he or she had not participated in simulated patient exercises prior to or during residency.

Of the obstetrical scenarios provided in the survey, the majority (>50%) of second-year residents felt uncomfortable counseling patients with the following issues: placenta accreta; those with a sentinel bleed from a placenta previa; pre-viable preterm premature rupture of membranes; and those patients requesting a vaginal birth after a cesarean delivery. These clinical issues were the basis for the four scenarios in our simulated patient exercise. Both pre-simulation on-line survey respondents (PGY2 and PGY1) selected case-based and simulation-based learning as the most effective learning method, and lectures as the least effective format for learning.

### First-year residents

Our first-year class is also comprised of ten residents, all of whom are female ranging from 24–31 years of age. Nine residents (90%) responded to our pre-session survey. Seven of the first-year residents (78%) stated that they were “somewhat comfortable” with discussing the risks and benefits of a medical decision. All respondents reported that they had participated in a simulated patient interaction prior to residency, with only one person reporting a negative experience. As seen in Table 2, none of the respondents (0%) reported that either lecture or journal club was an effective method by which to learn new material. All respondents selected case-based learning and simulations as the best ways to learn new material.

**Table 2:** Preferred learning style of first-year residents

**Learning style**	**Frequency N (%)**
Lectures	0 (0%)
Journal club	0 (0%)
Case-based teaching	4 (44%)
Discussion groups	3 (33%)
Simulation-based learning	2 (22%)

Seven first-year residents were able to participate in the “Intern Ed” session. All being satisfied with the experience as evidenced by the post-exercise survey, (found in Additional file 4) the first-year residents indicated that they would employ the strategies learned during the session in their daily practice of medicine. When asked the question *What do you think was most valuable thing that you learned from today’s session?,* one intern responded with the following: “I think it’s helpful to see other people model difficult conversations, so that I can incorporate different styles/strategies that work for me into my own practice”. Another intern stated that “the medical knowledge of PPROM and repeat C-section counseling” was most valuable. All respondents to the post-exercise survey (100%) reported that they would use the strategies that they have learned during the simulated patient exercise in future clinical encounters.

In further consideration of the post-exercise survey, the first-year residents appreciated the practical application of medical knowledge. While one intern stated, “I think the topics were covered were appropriate and helpful”, two interns responded that they wished that we had included a scenario on how to counsel patients with fetal loss. Two other interns also reported that they thought discussing patients with substance abuse/dependence and how physicians’ biases may influence the management of these patients. Three (43%) of the participated wished that they had more time to read the provided articles. Only one of the seven first-year residents participating indicated that he/she did not like role-playing, which was consistent with this individual’s past experience with this education technique.

When asked to provide feedback on the session, three interns stated that they had a positive experience with the simulation, with one reporting “I very much liked this as a learning tool”. Two others had no suggestions for feedback for the simulation. In reference to the pre-intervention articles provided, one intern stated, “That was an imposing reading list” and another wanted them to be sent earlier. These survey results indicate that the participants appreciated various aspects of the simulation and were able to obtain skills that could be used in clinical practice. However, some of the participants determined that the structure of the simulated patient exercise could be used for a variety of other clinical situations.

## Discussion and conclusion

To satisfy the multitude of learning objectives, OB/GYN training programs have come to rely on including structured lectures, formal intraoperative experience, discussion groups, journal clubs and “one-on-one teachable moments”. “Role-playing”, or simulated patient interactions, is an educational technique that allows for simultaneous assessment of multiple domains of competency. We implemented a simulated patient interaction session in order to teach our interns interpersonal communication skills. In order to create a richer experience, we based the simulated cases for our interns on cases that their peers, the second year residents, had encountered the year prior. Our post-session survey results support the use of this educational technique. All of the first-year residents participating in this exercise felt that this simulated patient interaction was beneficial for their education. In the era of work hour restrictions, educational experiences need to be relevant to the learner. Our session utilizing resident-identified clinical knowledge gaps and focusing on skills translatable to other patient-physician interactions was well received and valued by the participants.

It is important to understand the context in which our results were obtained. We implemented our educational tool during the first quarter of the intern year, a time during which a majority of our interns have completed at least one clinical rotation on an obstetrical service. Therefore, it is highly likely that these interns may not yet possess to the clinical acumen to handle such obstetrical scenarios presented during our simulation. Our simulation allows interns to become exposed to novel clinical situations in a safe, non-threatening environment in which effective communication skills can be learned, practiced and then implemented in actual patient encounters. Thus, after the completion of our simulation, the interns have a framework upon which to build for the entirety of their residency.

In an effort to establish more efficient guidelines for the assessment of resident achievement, the ACGME announced the Obstetrics and Gynecology Milestone Project in September 2013 [13]. The milestones represent key areas in which OB/GYN residents are expected to attain proficiency prior to graduation. Each milestone contains levels in order to represent the progression a resident should have from intern year to chief year [13]. Our educational intervention can be generalizable to other residency programs, as all residency training in obstetrics and gynecology in the United States must attain proficiency in the milestones of both professionalism and systems-based practice. Importantly, simulated patient exercises enable OB/GYN residents to refine their interpersonal/communication skills and develop their sense of professionalism in a safe, non-judgmental environment. Simulated patient interactions allow coached practice on how to interface with various types of patients, while expanding their medical knowledge base through interactive discussion with peers. The scenarios used in our intervention are commonly encountered in practice, and thus relevant to the practice of general obstetrics and gynecology.

Our study does have limitations. As a pilot study the small sample size may limit external validity of our results. It is also important to note that such a simulated patient exercise among colleagues may not perfectly reflect real-life clinical scenarios. However, the intention of our intervention was to provide participants with a framework upon which to build for actual patient interactions. Additionally, 43% of the first-year residents indicated that they would have preferred more time to read the pre-intervention articles provided to them. In planning the next education session, we anticipate sending our any supplementary material earlier with more explicit guidance on how this will be utilized in the session.

While we constructed a simulated patient exercise using obstetrical scenarios in order to teach effective communication skills, we assert that simulated patient interactions can be readily adapted to address other topics in obstetrics and gynecology, including those focusing on risk-benefit analysis, informed consent, and end-of-life discussions. Further investigation is warranted to determine resident retention of these skills and how well they translate to the bedside. Such assessment could be accomplished by recorded patient encounters, patient satisfaction surveys and patient interviews.

Given the success of our intervention, we anticipate its use with subsequent first-year classes and, possibly, the residency at large. Future research to assess residents’ acquisition and retention of core knowledge using this educational technique is necessary. It is our hope that providing a foundation in proper patient-physician communication during the first year of residency training will lead to a graduating resident who is competent in this domain and who eventually will be ready for independent practice.

## Abbreviations

OB/GYN: Obstetrics and gynecology; ACGME: Accreditation council of graduate medical education; MFM: Maternal-fetal medicine.

## Competing interests

The authors declare that they have no competing interests.

## Authors’ contributions

OMY conceived of the study, participated in the construction of the educational intervention and its implementation, participated in data collection and data analysis and drafted the initial manuscript. KP participated in the construction of the educational intervention and its implementation, participated in data analysis and made significant contributions to the writing of the manuscript. Both authors take full responsibility for the quality and the integrity of this manuscript. Both OMY and KP have approved the final version of this manuscript.

## Authors’ information

OMY is a clinical fellow in the Division of Maternal-Fetal Medicine at Magee-Women’s Hospital/University of Pittsburgh Medical Center in Pittsburgh, Pennsylvania. He is a member of the Association of Professors in Gynecology and Obstetrics.

KP is an assistant professor in the Division of Maternal-Fetal Medicine and the assistant residency program director at Magee-Women’s Hospital/University of Pittsburgh Medical Center in Pittsburgh, Pennsylvania. She is a member of the Association of Professors in Gynecology and Obstetrics.

## Supplementary Material

Additional file 1 Second-Year OB/GYN Resident Survey. Click here for file

Additional file 2 First-Year OB/GYN Resident Pre-Intervention Survey. Click here for file

Additional file 3 **OB/GYN Intern Education Simulated Patient Scenarios.** These are the clinical situations used for the simulated patient exercise. Each scenario allows the participants to take on either the role of the patient, the physician or another member involved in the medical decision-making. Each scenario allows the participants to more fully understand the spectrum of patients they will encounter in everyday practice. This includes the “angry” patient, issues of cultural competency, relationship dynamics and the influence of religious practice on health care. Click here for file

Additional file 4 First-Year OB/GYN Resident Post-Intervention Questionnaire. Click here for file
