# Supplementary material for: Training obstetrics and gynecology residents to be effective communicators in the era of the 80-hour workweek: a pilot study
Source: BMC Res Notes. 2014 Jul 17;7:455. doi: 10.1186/1756-0500-7-455 (PMC4105231; doi:10.1186/1756-0500-7-455)
Supplement: Additional file 1 — Second-Year OB/GYN Resident Survey. [file 1756-0500-7-455-S1.docx]

**SUPPLEMENT 1: SECOND-YEAR OB/GYN RESIDENT SURVEY**

1. Prior to the start of second year, how comfortable were you in counseling a Maternal-Fetal Medicine patients?
2. Very comfortable (0)
3. Somewhat comfortable (1)
4. Neutral (2)
5. Somewhat uncomfortable (3)
6. Very uncomfortable (4)
7. Prior to seeing a MFM patient in triage or on the floor, do you feel intimidated
8. Strongly agree (0)
9. Somewhat agree (1)
10. Neutral (2)
11. Somewhat disagree (3)
12. Strongly disagree (4)
13. My experiences as a first-year resident prepared me sufficiently for counseling MFM patients.
14. Strongly agree (0)
15. Somewhat agree (1)
16. Neutral (2)
17. Somewhat disagree (3)
18. Strongly disagree (4)
19. Which teaching style is the **MOST EFFECTIVE** to learn?
20. Lecture (0)
21. Journal Club (1)
22. Case based teaching (2)
23. Discussion groups (3)
24. Simulation-based learning (4)
25. Which teaching style is the **LEAST EFFECTIVE** way to learn?
26. Lecture (0)
27. Journal Club (1)
28. Case based teaching (2)
29. Discussion groups (3)
30. Simulation-based learning (4)
31. Have you used role playing ever in your education, even outside of residency?
32. Yes (1)
33. No (0)
34. If yes, when? ________________________________________________
35. If you answered yes to question 7, was the role play effective?   What was good? Bad?
36. Do you feel that you have adequate exposure to outpatient management of high risk obstetrical, aka maternal-fetal medicine or “MFM” patients?
37. Strongly agree (0)
38. Somewhat agree (1)
39. Neutral (2)
40. Somewhat disagree (3)
41. Strongly disagree (4)

1. Please rank the following clinical scenarios in terms of your confidence in being able to counsel a patient **INDEPENDENTLY** (aka, you can do this without the help of the attending or fellow)? (1 being most confident and 7 least confident)

___ Counseling a woman at 18 weeks who has experienced PPROM on all her management options

___ Management options for a woman at 28 weeks who has experienced PPROM

___ Counseling a G1P0 at 20 weeks with a cervical length of 17 mm

___ Discussing the risks and benefits of a repeat cesarean delivery versus trial of labor after cesarean delivery

___ Counseling a woman at 26 weeks with a heavy bleeding episode on management options after you have diagnosed her with a complete placenta previa

____ A G1P0 diabetic patient presents to your clinic at 36 weeks for a prenatal visit.  Her fetus is breech.  She desires an ECV.  You go through the chart and see that her ultrasound yesterday indicates an EFW of 4400g.   (

___ A woman has an ultrasound highly suspicious for a placenta accreta. She desires to keep her uterus.

1. Which additional obstetric topics do you find challenging from a patient counseling perspective?
