# Supplementary material for: Training obstetrics and gynecology residents to be effective communicators in the era of the 80-hour workweek: a pilot study
Source: BMC Res Notes. 2014 Jul 17;7:455. doi: 10.1186/1756-0500-7-455 (PMC4105231; doi:10.1186/1756-0500-7-455)
Supplement: Additional file 2 — First-Year OB/GYN Resident Pre-Intervention Survey. [file 1756-0500-7-455-S2.docx]

**SUPPLEMENT 2: FIRST-YEAR OB/GYN RESIDENT PRE-INTERVENTION SURVEY**

1. My experiences in medical school have prepared me sufficiently to counsel patients on complex medical issues.
2. Strongly agree (0)
3. Somewhat agree (1)
4. Neutral (2)
5. Somewhat disagree (3)
6. Strongly disagree (4)
7. You feel comfortable discussing with a patient the both risks and benefits of a medical decision. This is known as informed consent.
8. Strongly agree (0)
9. Somewhat agree (1)
10. Neutral (2)
11. Somewhat disagree (3)
12. Strongly disagree (4)
13. Which teaching style is the **MOST EFFECTIVE** to learn?
14. Lecture (0)
15. Journal Club (1)
16. Case based teaching (2)
17. Discussion groups (3)
18. Simulation-based learning (4)
19. Which teaching style is the **LEAST EFFECTIVE** way to learn?
20. Lecture (0)
21. Journal Club (1)
22. Case based teaching (2)
23. Discussion groups (3)
24. Simulation-based learning (4)
25. Do you have experience with role playing/simulated patients prior to residency?
26. Yes (1)
27. No (0)
28. If yes, when? ________________________________________________
29. If you answered yes to question 5, please briefly describe your experience.
30. Have you had a Labor Suite rotation this year:
    1. Yes
    2. No
