# Supplementary material for: Training obstetrics and gynecology residents to be effective communicators in the era of the 80-hour workweek: a pilot study
Source: BMC Res Notes. 2014 Jul 17;7:455. doi: 10.1186/1756-0500-7-455 (PMC4105231; doi:10.1186/1756-0500-7-455)
Supplement: Additional file 3 — OB/GYN Intern Education Simulated Patient Scenarios. These are the clinical situations used for the simulated patient exercise. Each scenario allows the participants to take on either the role of the patient, the physician or another member involved in the medical decision-making. Each scenario allows the participants to more fully understand the spectrum of patients they will encounter in everyday practice. This includes the “angry” patient, issues of cultural competency, relationship dynamics and the influence of religious practice on health care. [file 1756-0500-7-455-S3.docx]

**SUPPLEMENT 3: OB/GYN INTERN EDUCATION SIMULATED PATIENT SCENARIOS**

SCENARIO #1: “My body, my uterus, my birth”

Dr. Carrington: You are the OB/GYN who is covering for your partner, Dr. Colby, while he is in the operating room doing this amazing total abdominal hysterectomy. You have not had a chance to preview your charts for the day, but you do recall that Dr. Colby had said that a “special” patient, Ivana, was on your panel today. Dr. Colby received a phone call from Ivana yesterday asking about her delivery, but he told her that he would defer those answers to you. Ivana is waiting for you in your exam room.

SCENARIO #1: “My body, my uterus, my birth”

Ivana Veeback: Ivana, you are a 34 year old gravida 3 para 2002 currently at 33 weeks who has moved from the outskirts of Seattle to Pittsburgh. You have had 2 cesarean deliveries: one in 2008 for arrest of dilation at 9cm and one in 2010 for breech presentation. You had always wanted a vaginal delivery. Your previous OB/GYN practice has informed you that Dr. Colby may be willing to do that. You are frustrated but resigned to the fact that you have to talk to his partner, Dr. Carrington, today. You do not want any “drugs”, monitors or residents involved in your care. If challenged, you can become rather hostile.

___________________________________________________________

SCENARIO #2: “A Break Too Soon”

Dr. Salazar: You are urgently called to OB Triage for a consultation. The patient’s name is Marguerita Figueroa, a 28 year old gravida 2 para 1000 at 18+2 weeks gestation. Her private OB/GYN is not sure if she has broken her water. You are asked to examine her and to counsel her.

SCENARIO #2: “A Break Too Soon”

Marguerita Figueroa: You are a 28 year old gravida 2 para 1000 at 18+2 weeks gestation. You are sent to OB Triage by your private OB/GYN, who is not sure if you have broken your water. You have grown up in a religious household and are a devout churchgoer. Your first pregnancy was complicated by a shoulder dystocia in Mexico City for 7 minutes that resulted in death of the neonate on day of life #2.

_________________________________________________________________-

SCENARIO #3: “The Hospital Is Your New Home”

Dr. Russell: You are the resident who is the gatekeeper of OB Triage on a busy Friday night. You are running around and trying to coordinate the management of 4 patients, 3 of which are probably going home. Your 4^th^ patient, Ms. Lemke, needs your attention now. As you open the door, you notice that the bed sheets are covered in blood. She is accompanied by her husband.

SCENARIO #3: “The Hospital Is Your New Home”

Ms. Lemke: You are a 37 year old gravida 2 para 1001 at 26 weeks gestation, who is traveling with her husband from Illinois for vacation along with your 4 year old daughter. You really wanted to see the giant duck near Point State Park. You were diagnosed with a placenta previa on an 18 week ultrasound. While walking around the Point, you noticed your pants to be stained. Once back at the hospital, you noticed steady vaginal bleeding and were immediately taken to Magee by your husband. You really want to go back to Chicago.

SCENARIO #3: “The Hospital Is Your New Home”

Mr. Lemke: You are with your wife, Mrs. Lemke. She is a 37 year old gravida 2 para 1001 at 26 weeks gestation with a placenta previa. You are a high-power executive in Chicago who is on vacation with your wife and 4 year old daughter in Pittsburgh. You are not happy about this. You have taken your wife to Magee because she is bleeding. You need to be back in Chicago tomorrow for a board meeting. You hate hospitals.

______________________________________________________________________

.

SCENARIO #4: “When Your Placenta Doesn’t Want To Say Goodbye”

Dr. Grant: You are about to talk to your patient, Monica Clarkson. Monica is a 29 year old gravida 1 para 0 at 27 weeks gestation with type 1 diabetes. Monica is a Jehovah’s Witness. You obtained a growth scan per protocol. The ultrasound report stated the following: estimated fetal weight of 875 grams (50^th^ percentile for gestational age), anterior placenta, and an amniotic fluid index of 126 mm. There are numerous placental lakes and a 1.9 cm area of loss of demarcation between the anterior myometrium and posterior bladder, concerning for placenta accreta.

SCENARIO #4: “When Your Placenta Doesn’t Want To Say Goodbye”

Monica Clarkson: You are a 29 year old gravida 1 para 0 at 27 weeks gestation with type 1 diabetes. You are a Jehovah’s Witness and will never accept a blood transfusion. You desire to have at least 3 children, and this is not negotiable. You are waiting for Dr. Grant to come talk with your regarding the results of today’s growth ultrasound.
