# Supplementary material for: Training obstetrics and gynecology residents to be effective communicators in the era of the 80-hour workweek: a pilot study
Source: BMC Res Notes. 2014 Jul 17;7:455. doi: 10.1186/1756-0500-7-455 (PMC4105231; doi:10.1186/1756-0500-7-455)
Supplement: Additional file 4 — First-Year OB/GYN Resident Post-Intervention Questionnaire. [file 1756-0500-7-455-S4.docx]

**SUPPLEMENT 4: FIRST-YEAR OB/GYN RESIDENT**

**POST-INTERVENTION QUESTIONNAIRE**

1. What do you think was most valuable thing that you learned from today’s session?
2. Will you use the strategies that you learned here in your daily practice of medicine?

________________________________________________________________

1. What clinical or non-clinical topic would you have liked to have been included in today’s session?
2. What suggestions do you have for improving the session?

________________________________________________________________
